# TEMPORAL TRENDS IN INCIDENCE RATES OF VISION IMPAIRMENT AMONG MIDDLE-AGED AND OLDER CHINESE

**DOI:** 10.1093/geroni/igz038.1803

**Published:** 2019-11-08

**Authors:** Gui-Ying Cao, Shan-Shan Yao, zi-shuo Chen, Zi-Ting Huang, Beibei Xu

**Affiliations:** 1 Department of Epidemiology and bio-statistics School of Public Health, Peking University, Beijing, China; 2 Peking University Medical Informatics Center, Beijing, China

## Abstract

This study aimed to investigate temporal trends in the incidence rates (IRs) of vision impairment (VI) and explore its associated factors among middle-aged and older Chinese. Data were obtained from China Health and Retirement Longitudinal Study (CHARLS, 2011-2015) and Chinese Longitudinal Healthy Longevity Survey (CLHLS, 1998-2014). There were 20,467 participants with a mean age of 57.4 years from CHARLS and 21,210 participants with a mean age of 90.0 years from CLHLS. Distance and near vision were self-reported in CHARLS and performance-based vision was assessed in CLHLS. Crude and age-adjusted IRs per 1,000 person years (PY) of distance vision impairment (DVI), near vision impairment (NVI) and both distance and near vision impairment (DNVI) in CHARLS and VI in CLHLS were calculated for each wave of surveys. Risk factor adjusted temporal trends in the incidence were examined using regression models. Age-standardised IRs for DVI, NVI and DNVI significantly decreased from 79.4 to 41.2, 95.1 to 48.0 and 40.0 to 21.3 (P < 0.001) from 2013 to 2015, respectively. Male gender, higher education, no ADL disability, no depression, no multimorbidity were associated with lower IRs of DVI, NVI or DNVI. Younger age was associated with lower IRs of DVI and DNVI but higher IRs of NVI. Age-standardised IR of VI significantly decreased from 131.6 to 71.9 (P = 0.010) from 2000 to 2014, and higher education and living in the South were associated with lower IR of VI. Future studies may further investigate the causality of such phenomenon.

